# Assessing Feeding Damage from Two Leaffooted Bugs, *Leptoglossus clypealis* Heidemann and *Leptoglossus zonatus* (Dallas) (Hemiptera: Coreidae), on Four Almond Varieties

**DOI:** 10.3390/insects10100333

**Published:** 2019-10-07

**Authors:** Andrea L. Joyce, Apurba K. Barman, David Doll, Bradley S. Higbee

**Affiliations:** 1 Department of Public Health, University of California Merced, 5200 N. Lake Road, Merced, CA 94343, USA; 2 Department of Entomology, University of Georgia, Tifton, GA 31793, USA; abarman@uga.edu; 3 University of California, Cooperative Extension (UCCE), Merced, CA 95341, USA; dadoll@ucanr.edu; 4 Trece, Inc. 13404 W. Anapama Dr., Sun City West, AZ 85375, USA; bhigbee@trece.com

**Keywords:** insect feeding damage, economic damage, leaffooted bug, almonds, pistachios, citrus, pomegranate

## Abstract

Leaffooted bugs (*Leptoglossus spp*; Hemiptera: Coreidae) are phytophagous insects native to the Western Hemisphere. In California, *Leptoglossus clypealis* and *Leptoglossus zonatus* are occasional pests on almonds. Early season feeding by *L. clypealis* and *L. zonatus* leads to almond drop, while late season feeding results in strikes on kernels, kernel necrosis, and shriveled kernels. A field cage study was conducted to assess feeding damage associated with *L. clypealis* and *L. zonatus* on four almond varieties, Nonpareil, Fritz, Monterey, and Carmel. The objectives were to determine whether leaffooted bugs caused significant almond drop, to pinpoint when the almond was vulnerable, and to determine the final damage at harvest. Branches with ~20 almonds were caged and used to compare almond drop and final damage in four treatments: (1) control branches, (2) mechanically punctured almonds, (3) adult *Leptoglossus clypealis*, and (4) adult *Leptoglossus zonatus*. Replicates were set up for eight weeks during two seasons. Early season feeding resulted in higher almond drop than late season, and *L. zonatus* resulted in greater drop than *L. clypealis*. The almond hull width of the four varieties in the study did not influence susceptibility to feeding damage. The final damage assessment at harvest found significant levels of kernel strikes, kernel necrosis, and shriveled almonds in bug feeding cages, with higher levels attributed to *L. zonatus* than *L. clypealis*. Further research is warranted to develop an Integrated Pest Management program with reduced risk controls for *L. zonatus*.

## 1. Introduction

Leaffooted bugs in the genus *Leptoglossus* Guérin-Méneville (Hemiptera: Coreidae) are large phytophagous insects native to the Western Hemisphere. At least 61 species are known [1], and several species are pests in forests or agricultural systems [2,3,4,5,6,7]. Many *Leptoglossus* spp. are multivoltine, which allows them to exploit multiple hosts per year [8]. Direct damage to crops is caused when *Leptoglossus* spp. feed by probing their stylets into fruits and seeds, and secondary damage can occur through the transmission of pathogens at the feeding site [6,9,10]. Field studies assessing the feeding damage of insects can provide information about the phenology of the pest and pinpoint when during the growing season insect feeding occurs, as well as determine when the crop is susceptible to damage or losses [11,12,13,14].

Two species of *Leptoglossus*, *Leptoglossus clypealis* Heidemann and *Leptoglossus zonatus* (Dallas) are occasional pests feeding on almond and pistachio crops in the Central Valley of California [2,3,11,15,16,17,18]. *L. clypealis* was considered to have a more limited distribution in the western United States [19,20], but is now reported to occur through the Midwest into Illinois, with some additional records from the east coast [21]. While *L. clypealis* is noted in California for infesting almonds and pistachios, it has been recorded from at least twenty host plants throughout its range [18]. *L. zonatus* is found in much of the Western Hemisphere ranging from Brazil into the southern United States [4,20,22] on a wider range of host plants including citrus, pomegranates, almonds, and corn, among others [6,18,23]. In California, *Leptoglossus spp.* are reported to overwinter in adult aggregations [24]. As temperatures warm up in the spring, the adults disperse from aggregations and can be observed in almond orchards. Feeding by *L. zonatus* and *L. clypealis* on almonds results in clear sap exuding from developing fruit, known as gummosis [25]. Early season feeding by these two species in March and April can result in almond drop, while feeding later in the growing season can directly damage almond kernels and result in losses [16,17]. Both *L. zonatus* and *L. clypealis* are reported to be more abundant in the last few years, perhaps due to increased plantings of almonds in California [17,26]. Approximately 1.36 million acres of almonds were cultivated in California in 2017, with an estimated value of $5.6 billion [27]. Determining the level of damage from feeding *L. clypealis* and *L. zonatus* during the growing season in a field experiment will help determine the relative damage from each of these insects, and demonstrate when the almond crop is most vulnerable to *Leptoglossus* feeding, which in turn will help to determine the timing of prevention and control measures. The objectives of this work were to (1) determine the level of almond drop from feeding by adult *L. clypealis* and by *L. zonatus*, (2) compare how almond drop varies during the growing season, (3) consider almond size and its relationship to feeding damage, and (4) quantify the final damage to almonds at harvest time from feeding by *L. clypealis* and *L. zonatus*.

## 2. Methods

### 2.1. Field Sites

The four almond varieties used in this study were Nonpareil, Fritz, Monterey and Carmel. An orchard in Winton, Merced County, California (37°22′45.73″ N, 120°37′39.82″ W), had Nonpareil and Fritz varieties, while a second orchard in Merced, Merced County, California (37°18′10.63″ N, 120°23′18.14″ W), had Monterey and Carmel varieties. Studies were conducted on farms with permission from the owners. The study was run over two growing seasons, from late March through August in 2014 and in 2015.

#### 2.1.1. Insect Colonies

Colonies of *L. clypealis* and *L. zonatus* were maintained in the laboratory and were used for field cage trials to assess feeding damage. The insect populations used to start the colonies were collected from almond and pomegranate orchards in Manteca, Ripon and Lost Hills, California. Colonies were maintained in insect rearing cages (61 × 61 × 91 cm) (Bioquip, Rancho Dominguez, CA, USA) with a diet of fresh green beans and corn on the cob, and raw peanuts, sunflower seeds, pine nuts, and pumpkin seeds. Each cage was also provisioned with 1–3 arborvitae plants (*Thuja occidentalis*) in 1 gallon containers which served as habitat and a water source for the insects. Food was replaced at least once a week. Colonies were maintained year-round under laboratory conditions at 27 °C and a 14:10 L:D cycle.

#### 2.1.2. Experimental Set Up

##### *Leptoglossus* Feeding and Almond Abscission, and Final Damage Assessment

The effect of adult *L. clypealis* and *L. zonatus* feeding was evaluated on four almond varieties (Nonpareil, Fritz, Monterey and Carmel) during the growing season from the end of March until mid-August. The four experimental treatments included (1) controls, (2) mechanical damage to the developing almonds, (3) feeding by adult *L. clypealis* and (4) feeding by adult *L. zonatus*. All four treatments included an almond branch with approximately 20 almonds, covered by a sleeve cage consisting of a 5-gallon organdy mesh paint strainer (Blue Hawk, Trimaco LLC, Morrisville, NC, USA) closed with a large binder clip. Control branches with almonds served to determine the natural level of almond abscission (drop) during the growing season. The second treatment consisted of branches with almonds which were mechanically punctured to mimic feeding damage caused by the insect stylet (mouthpart) probing into developing nuts. Each developing almond was punctured 4–5 times with a #1 insect pin (Bioquip, Rancho Dominguez, CA, USA). Puncturing almonds served an additional purpose, which was to provide an estimate of the time of shell hardening; shells typically became resistant to puncture by the end of April. The third treatment consisted of 5 adult *L. clypealis* (3 females/2 males) which were allowed to feed for 4–6 d and were then removed. The fourth treatment was similar to the third but used 5 adult *L. zonatus* (3 females/2 males). For these treatments, insects were taken from the lab colony, and were first isolated with only water (no food) for 24 h before placing them into an experimental sleeve-cage. Each week in each almond variety, four branches were setup as controls, four were setup with punctured almonds, one branch each was setup with *L. clypealis,* and one with *L. zonatus.* For approximately eight weeks, the four treatments were replicated in the same manner on new trees in each of the four almond varieties. For Monterey and Carmel varieties in 2014, fields could not be entered on two weeks due to flood irrigation, and this resulted in six weeks of observations rather than 8. In 2015, the same experiment was repeated on the same four varieties, with the exception that feeding damage was assessed for *L. zonatus* but not for *L. clypealis*, due to an insufficient numbers of adult *L. clypealis* to complete the experimental replicates.

#### 2.1.3. Data Collection and Analyses

##### Almond Drop in Four Varieties

Each week, data were recorded on the number of almonds fallen (abscised) from branches within all cages that were setup in previous weeks. These data were used to determine the mean percent almond drop in each of the four treatments for each of the four varieties. An analysis of variance test was considered to compare means, but data were not normally distributed, even after log transformation. Thus, nonparametric Kruskal–Wallis (KW) tests were used as they do not assume a distribution for the data [28]. Post-hoc pairwise comparisons were by Steel–Dwaas tests and were considered significant if *p* < 0.05 [29]. In 2015, similar comparisons were made for the mean almond drop for three treatments (control, puncture, *L. zonatus*) within each almond variety.

To examine when almonds were most susceptible to almond drop from feeding by each *Leptoglossus* species, the mean percent almond drop was compared among the experimental weeks within each bug feeding treatment. A Chi-square goodness of fit test was used to first examine whether the percent almond drop from a *Leptoglossus* species was equal among the weeks of the study for each almond variety. If almond maturity had no impact on insect feeding damage, the percent drop would be equal among weeks of the study. When a significant difference among weeks was observed, subsequent tests were by Fisher’s Exact tests to compare pairs of weeks (e.g., week 2 vs. week 3 for almond drop from *L. clypealis*).

### 2.2. Almond Size and Hull Width

Hull width could influence whether an almond variety is more susceptible to feeding damage. Hull width was determined during the weeks of observations in both 2014 and 2015. Each week, 10–15 almonds were collected in each of the four varieties and measured to determine almond size (length and width) and hull width (mm). The mean almond length, almond width, and hull width were each compared among the four varieties using the non-parametric Kruskal–Wallis (KW) test, as data were not normally distributed. Pairwise comparisons were done using Steel–Dwass tests [29].

### 2.3. Final Damage Assessment

Just before harvest, the almonds remaining in field cages were removed to conduct a final damage assessment. For each control and each branch with mechanically damaged almonds, a subsample of four almonds was used to assess several damage parameters. For branches caged with *L. clypealis* or *L. zonatus*, all remaining almonds were removed and used for the final damage assessment. Four parameters of feeding damage were determined, hull strikes, almond kernel necrosis, strikes on the kernel, and shriveled kernels. A strike on the hull was characterized by a black or brown spot. Almond kernel necrosis was indicated by a kernel with brown or black necrotic areas. A strike (a mark or line) on the kernel was the third type of damage. The fourth and final damage type was whether or not the almond kernel was shriveled. Damage was recorded for each category as presence or absence. Data were summarized separately for each of the two years. For each year, a Chi square goodness of fit test was used to examine whether or not there were equal levels of a damage type among three treatment categories (e.g., controls, *L. clypealis*, *L. zonatus*) within a variety. If there was a significant difference in damage among the three treatments in a variety, then Fisher’s Exact tests were used to compare each pair of treatments. Tests were considered significant at the *p* < 0.05 level.

## 3. Results

### 3.1. Total Almond Abscission from Leptoglossus Feeding in 2014

**Figure 1:**
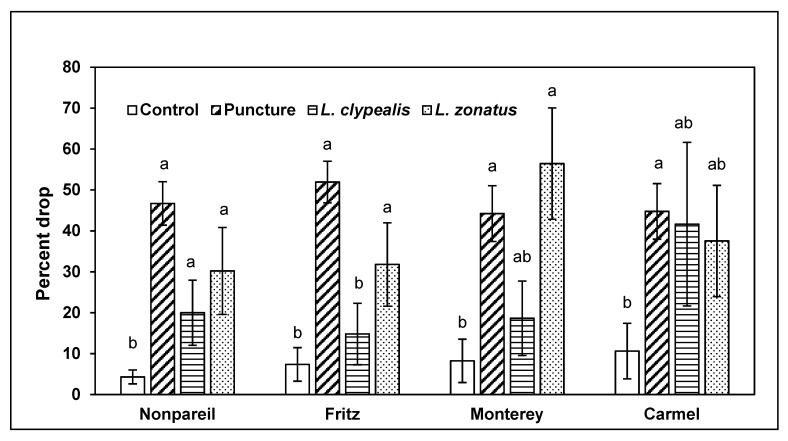
Mean (±SE) almond drop in four experimental treatments in four almond varieties in 2014. Within each variety, there was a significant difference among treatments (Kruskal–Wallis). Pairwise comparisons were by Steel–Dwaas tests (*p* < 0.05), and significant differences are indicated by different letters.

Almond drop varied across treatments in all four varieties (Nonpareil (NP), KW: χ^2^ = 15.75; df = 3; *p* = 0.001; Fritz (F), KW: χ^2^ = 25.74; df = 3; *p* < 0.001; Monterey (M), KW: χ^2^ =12.46; df = 3; *p* = 0.006; Carmel (C), KW: χ^2^ = 11.05; df = 3; *p* = 0.011). In Nonpareil, controls had less abscission than branches with mechanically punctured almonds, or those where *L. clypealis* or *L. zonatus* had fed (Figure 1). On Fritz, drop in the controls was similar to *L. clypealis*, and both were lower than the *L. zonatus* and the puncture treatments. In Monterey, *L. zonatus* and the punctured treatment were higher than the control while *L. clypealis* was intermediate. Finally, in Carmel, drop levels were highest in cages with punctured almonds, but almond drop did not vary significantly between bug feeding cages and the control (Figure 1).

### 3.2. Total Almond Abscission from Leptoglossus Feeding in 2015

**Figure 2:**
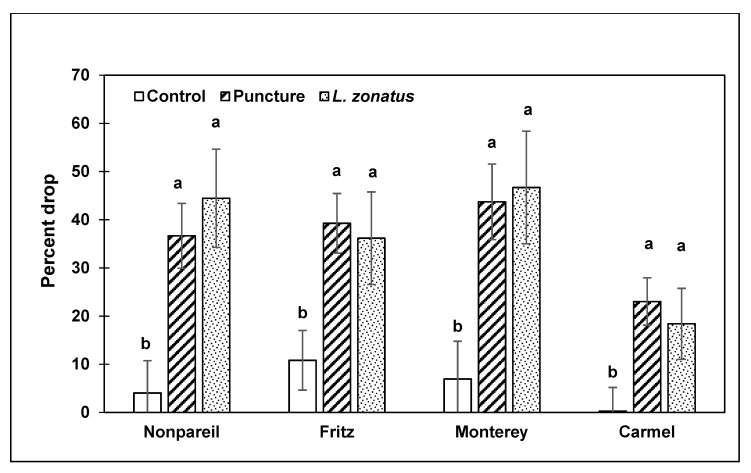
Mean (±SE) percent almond drop from three treatments in four almond varieties in 2015. Within each variety, a significant difference in treatments was found (KW, *p* < 0.05). Pairwise comparisons were by Steel–Dwaas tests (*p* < 0.05), and significant differences are indicated by different letters.

In 2015, three experimental comparisons included control branches, mechanically punctured almonds, and cages with adult *L. zonatus*. In Nonpareil (NP), Fritz (F), Monterey (M) and Carmel (C), there was a significant difference in almond drop among the three treatments (NP, KW: χ^2^ = 10.10; df = 2; *p* = 0.006; (F), KW: χ^2^ = 7.16; df = 2; *p* = 0.03; (M), KW: χ^2^ = 7.29; df = 2; *p* = 0.026; (C), KW: χ^2^ = 14.89; df = 2; *p* < 0.001) (Figure 2). In Nonpareil, Fritz and Monterey, cages with punctured almonds and those with *L. zonatus* had higher levels of almond drop than controls (Figure 2).

### 3.3. Comparisons of Almond Abscission by Week

#### 3.3.1. *L. clypealis* in 2014

In Nonpareil, there was a significant difference in almond drop from *L. clypealis* feeding during the eight weeks of observations (χ^2^ = 177.50, df = 7, *p* < 0.001) (Table S1). Almond drop was significantly higher in the second and third weeks than in the other six weeks (all *p* < 0.05; Tables S1 and S2). In Fritz, almond drop from *L. clypealis* also varied significantly among the eight weeks (χ^2^ = 223.33, df = 7, *p* < 0.001), and week three was higher than all other weeks (*p* < 0.001; Table S2).

Observations in Monterey and Carmel were obtained for six weeks. Eight weeks of observations were planned, but orchards were flooded on two of the weeks (week 1 and 7), which restricted entry (Table S1). In Monterey, almond drop associated with *L. clypealis* varied significantly among the weeks of observations (χ^2^ = 133.26, df = 5, *p* < 0.001). Week 2 and 3 had higher levels of drop than the other weeks (*p* < 0.05, Table S2). In Carmel, almond drop varied significantly among the weeks (χ^2^ = 287.02, df = 5, *p* < 0.001); week 5 and 6 were significantly greater than others (*p* < 0.05; Table S2).

#### 3.3.2. *L. zonatus* in 2014

Almond abscission from *L. zonatus* in NP varied significantly among observation weeks (χ^2^ = 319.37, df = 7, *p* < 0.001); drop in week three was greater than in other weeks (*p* < 0.05; Tables S1 and S3). In Fritz, almond drop varied significantly among the weeks (χ^2^ = 269.15, df = 7, *p* < 0.001); week 2 and week 3 were significantly higher than in other weeks (*p* < 0.05; Tables S1 and S3). Both Monterey and Carmel overall had significant variation among the weeks of observations [(M), χ^2^ = 183.41, df = 5, *p* < 0.0001; (C), χ^2^ = 150.55, df = 5, *p* < 0.001]. In Monterey, *L. zonatus* feeding in week 2, 3, 5 and 6 were higher than almond drop in week four and eight (*p* < 0.05). In Carmel, week three and five drop were higher than in weeks 2, 4, 6 and 8 (*p* < 0.05; Tables S1 and S3).

#### 3.3.3. *L. zonatus* in 2015

In 2015, almond drop from *L. zonatus* feeding varied significantly among the weeks of the study in Nonpareil and Fritz (NP, χ^2^ = 319.72, df = 8, *p* < 0.001; F, χ^2^ = 244.86, df = 8, *p* < 0.001). In Nonpareil, weeks 1–2 and 4–6 had significantly higher levels of drop than in weeks 7–9 (*p* < 0.05; Tables S4 and S5). Almond drop in Fritz was higher in week 2 than in other weeks (*p* < 0.05; Tables S4 and S5) and drop in week 5 was greater than in weeks seven to nine (*p* < 0.05; Table S5). In Monterey, significant variation occurred among the weeks (χ^2^ = 336.28, df = 8, *p* < 0.001); drop in week three was higher than in week 4 and 6–9 (*p* < 0.05; Tables S4 and S5). Finally, Carmel almond drop varied significantly as well (χ^2^ = 359.42, df = 8, *p* < 0.001). Week five almond drop was higher than in each of the other weeks (*p* < 0.05) (Tables S4 and S5).

### 3.4. Almond Size Parameters in 2014 and 2015

**Figure 3:**
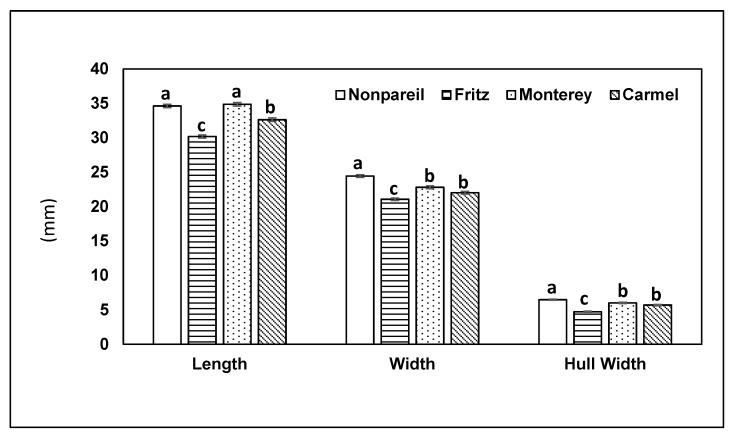
Mean (±SE) almond length and width, and hull width, in four almond varieties in 2014. Overall test within each parameter is by Kruskal–Wallis, and pairwise comparisons are by Steel–Dwass tests (*p* < 0.05). Within each parameter, columns with the same letters do not differ significantly.

In 2014, mean almond length was significantly different among the four almond varieties (KW: χ^2^ = 28.65, df = 3, *p* < 0.001). Nonpareil and Monterey were significantly longer than Fritz and Carmel (Figure 3), Carmel were intermediate in length and Fritz were the shortest (Figure 3). Almond width also varied among the four varieties (KW: χ^2^ = 110.97, df = 3, *p* < 0.001). Nonpareil were widest, Carmel and Monterey were intermediate, and Fritz least wide (Figure 3). Hull width (thickness) also varied significantly (KW: χ^2^ = 123.11, df = 3, *p* < 0.001); the hull of Nonpareil was thickest, the hull width of Monterey and Carmel were intermediate, and Fritz had the narrowest hull width (Figure 3).

**Figure 4:**
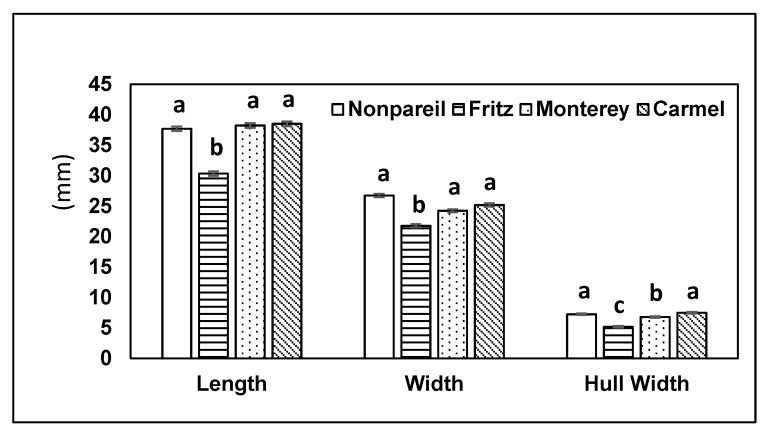
Mean (±SE) for three size parameters in 2015. Each parameter was compared by a Kruskal–Wallis test, and pairwise comparisons were by Steel–Dwass (*p* < 0.05). Within each parameter, columns with the same letters do not differ significantly.

The almond sizes in 2015 followed a pattern similar to 2014. The length of Nonpareil, Monterey and Carmel varieties were similar and significantly longer than Fritz (KW: χ^2^ = 117.56, df = 3, *p* < 0.001) (Figure 4). The width of the fruit in four varieties differed significantly (KW: χ^2^ = 152.88, df = 3, *p* < 0.001); Nonpareil was widest followed by Carmel and Monterey, and then Fritz (Figure 4). The hull width of the four almond varieties varied significantly (KW: χ^2^ = 111.73, df = 3, *p* < 0.001). Carmel and Nonpareil had thicker hulls, Monterey was intermediate, and hull width was thinnest in Fritz (Figure 4).

### 3.5. Final Damage Assessment in 2014

**Figure 5:**
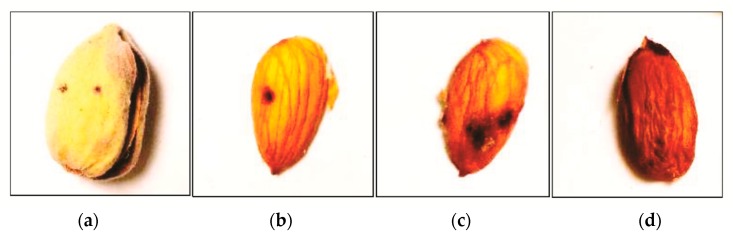
Four types of almond damage evaluated at harvest included (**a**) hull strikes, (**b**) nut strikes, (**c**) kernel necrosis, and (**d**) shrived kernels.

In Nonpareil, three of the damage parameters (hull strike, kernel necrosis, and nut strikes) varied significantly among the controls and bug feeding treatments (*p* < 0.001, Table 1, Figure 5a–d). For all three parameters, *L. clypealis* and *L. zonatus* had higher damage levels than the controls, and there was no difference between the *L. clypealis* and *L. zonatus* (*p* < 0.05) (Table 1). However, the percent of almond kernels shriveled at harvest was not significantly different between the controls and the bug feeding treatments (χ^2^ = 1.20, df = 2, *p* = 0.55) (Table 1).

In the variety Fritz, the percent of almonds with hull strikes, kernel necrosis, and nut strikes again varied among the controls and *Leptoglossus* treatments (Table 1). For hull strikes, both *Leptoglossus* species produced a higher percentage of hull strikes than controls (*p* < 0.05) (Table 1). Kernel necrosis was lowest in controls, intermediate in *L. clypealis* and highest in *L. zonatus* (*p* < 0.05; Table 1. A similar pattern was observed for nut strikes in Fritz (*p* < 0.001) (Table 1). The percent of shriveled kernels was low in all three treatments and did not differ significantly between the controls and the bug feeding treatments (χ^2^ = 1.0, df = 2, *p* = 0.61) (Table 1).

In Monterey, hull strike was significantly higher in the cages with *L. clypealis* and *L. zonatus* than in the controls (*p* = 0.012; Table 1). Nut strike damage was also higher in bug treatments than in the control (*p* < 0.05; Table 1). Percent kernel necrosis varied among the treatments (*p* < 0.001, Table 1); necrosis was higher from *L. zonatus* than the two other treatments (Table 1). Finally, shriveled kernels were higher in the *L. zonatus* treatment than in the control or the cages with *L. clypealis* (*p* < 0.05, Table 1), and the level of damage in controls and in *L. clypealis* cages was not significantly different.

Almonds in Carmel trees had nearly twice the level of hull strikes from *L. zonatus* as from *L. clypealis*, and both were higher than in controls (*p* < 0.05, Table 1). Kernel necrosis was higher in *L. zonatus* than the other two treatments and did not differ between the control or the *L. clypealis* cages (*p* < 0.05). Percent nut strike was also higher in cages with *L. zonatus* than in *L. clypealis* and the controls (*p* < 0.05. Finally, shriveled kernels varied among treatments and were similarly higher in the *L. zonatus* cages than in the *L. clypealis* cages or the controls (*p* < 0.05, Table 1).

### 3.6. Final Damage Assessment in 2015

In Nonpareil in 2015, all four damage parameters were higher in the *L. zonatus* cages than in the controls (all *p* < 0.001, Table 2). In Fritz, all damage parameters with the exception of shriveled kernels were higher in *L. zonatus* cages compared to the control (*p* < 0.001, Table 2). In Monterey, all four damage parameters were higher in *L. zonatus* cages than control cages (*p* < 0.001, Table 2). In Carmel, hull strike, kernel necrosis, and almond nut strikes were higher in *L. zonatus* treatments than controls (*p* < 0.001, Table 2). However, there was no significant difference in Carmel between the *L. zonatus* cages and the control for the percent of kernels shriveled (*p* = 0.45, Table 2).

## 4. Discussion

This study examined the impact from feeding by two *Leptoglossus* species, *L. clypealis* and *L. zonatus,* on developing almonds in four varieties during the growing season. Almonds of each variety were caged with equal numbers of adults of each species to determine the potential impact. Early in the growing season, leaffooted bugs fed by puncturing their stylets into the developing almond accessing the jelly-like immature kernel. In both years, almond drop in the controls was low, while almond drop in cages with *Leptoglossus* adults was twice as high. The first month of the study in April had higher levels of almond drop than in the later weeks of the study in May. The time in April corresponded to weeks when the almond shell could be punctured mechanically and indicated that the developing almonds were more vulnerable than in May, when the shell had hardened. Overall, almond drop during the season was lower from *L. clypealis* than from *L. zonatus*. This may be due to the smaller size of *L. clypealis* compared to *L. zonatus* adults. Monterey had higher levels of almond drop from *L. zonatus* in both years than the other three varieties. Feeding by leaffooted bugs also caused significant damage to almonds in the form of nut strikes, kernel necrosis and shriveled kernels. The final damage observed at almond harvest was higher overall from feeding associated with *L. zonatus* than *L. clypealis.*

Other studies have found significant crop damage from the presence of *Leptoglossus clypealis* and *Leptoglossus zonatus.* For example, feeding by *L. zonatus* in physic nut increased fruit abortion [12]. A study of *L. clypealis* on pistachio found that when one adult *L. clypealis* fed for 48 h, the number of fruits dropped was higher on average than in controls [11]. *L. zonatus* feeding in citrus resulted in spots on the outer citrus rind and feeding on satsuma mandarin by 1–3 adults for 14 days resulted in 37.5–100 percent of premature fruit abortion [6]. Studies such as these demonstrate the need for controls, to determine the natural level of fruit abscission, and which damage symptoms can be attributed to insect feeding.

### 4.1. Overall Almond Drop by Observation Week

Developing almonds may be more susceptible than mature almonds to feeding damage from Hemiptera, as has been observed in pistachios [3]. In the present study, the almond shell could be punctured by an insect pin until the end of April but by May, the external almond shell became too firm to puncture and almonds were presumed no longer susceptible to bug feeding. The majority of almond drop in the four varieties occurred in the first few weeks of observations. After the fifth observation week of caging *Leptoglossus* on branches, the percent almond drop decreased relative to the early weeks of observations and remained minimal. This agrees with an early study by Haviland [16], where almonds were artificially damaged by puncturing, and more almond drop occurred in April than in May. Our study was a no-choice test, where bugs were caged with only one almond variety; when offered one variety and no choice, the levels of almond drop were similar among the four varieties. However, in a natural field-study where almond drop was assessed after a *Leptoglossus* infestation, higher levels of almond drop were observed in Fritz [16]. When provided with a choice of varieties, *L. clypealis* appeared to prefer to feed on Fritz.

Almonds on the trees that were fed on by bugs later in their development may not drop but can suffer downgrades or rejects at harvest as observed for other crops [16]. In pistachios, *Leptoglossus* sp. was capable of causing feeding damage to the pistachio kernel even after the hardening of the shell [30]. Michalides et al. [31] found that the large bugs including *Leptoglossus clypealis* were able to cause pistachio kernel damage until late June, when nut development was at the final stage [3]. A late season feeding study of large bugs in pistachios found that kernel necrosis could range up to 20% [15]. The vulnerability of almonds later in the growing season (after May) has not been fully investigated. *Leptoglossus* may cause feeding injury to almonds which are well developed, and thus might be monitored and controlled throughout the growing season.

In other systems, fruit size and shell hardness has been found to influence the level of insect feeding damage. Feeding on physic nut by *Leptoglossus zonatus* resulted in larger fruits having more seed abortion and feeding resulted in more shriveled or damaged nuts [12]; similarly, some varieties of olives with larger fruits had a higher infestation rate by the olive fruit fly, *Bactrocera oleae* [32]. In hazelnut, shell thickness was not correlated with damage by brown marmorated stink bugs, *Halyomorpha halys*, and the authors suggested that shell thickness should not be a criterion to select cultivars resistant to *H. halys* [33]. Follett et al. [34] reported that husk and shell thickness in macadamia nut do not primarily determine susceptibility of fruits to *Nezara viridula*.

### 4.2. Almond Size and Leptoglossus Feeding Damage

Hull width could relate to the susceptibility of the almond to feeding damage by the two leaffooted bug species [16]. Although there was a significant difference in hull widths between Nonpareil and Fritz, there was no relationship between hull width and almond drop. In addition, the hull width of Nonpareil was significantly thicker than Fritz, but hull strikes on Nonpareil and Fritz from *L. clypealis* and *L. zonatus* did not vary significantly, and nut strikes were also not higher on Fritz. This suggests that damage from *Leptoglossus* feeding on these two varieties was not influenced by hull width (i.e., almond size). In 2015, higher levels of damage were observed in all four varieties compared to 2014, but again did not relate to the hull width differences (Table 1 and Table 2).

### 4.3. Final Damage Assessment at Harvest

The final assessment of damage from *L. zonatus* and *L. clypealis* included hull strikes, nut strikes, kernel necrosis and shriveled kernels. Insects probing the hull results in hull strikes and suggest that the internal almond kernel may be damaged. Nut strikes, almond kernel necrosis and shriveled kernels can lead to downgrades or unsellable product. In both years of this study, the cages with both *Leptoglossus* species typically had higher levels of hull strikes and nut strikes than in the control cages. In 2014, *L. clypealis* feeding did not result in significant kernel necrosis compared to controls in any variety, but *L. zonatus* had higher levels of kernel necrosis than controls in Monterey and Carmel; in 2015, kernel necrosis was consistently higher in *L. zonatus* cages than in controls. In 2014, the percent of shriveled kernels at harvest did not vary between the controls and the *L. clypealis* cages in any variety. However, the percent of shriveled kernels was higher in *L. zonatus* cages than controls in two varieties, Monterey and Carmel, while in 2015, the cages with *L. zonatus* resulted in higher levels of shriveled kernels in Nonpareil and Monterey. Research has similarly found that insect feeding damage varies among almond varieties as well as in varieties of other crops such as in apple, blueberry and olives [13,17,32,35].

Hull strikes that are characterized by a spot on the external portion of the almond could correspond directly to internal damage such as nut strikes or almond damage. The number of external hull strikes was similar to the number of internal nut strikes and damaged nuts for *L clypealis.* However, for *L. zonatus* in both years, the number of hull strikes recorded was often lower than the number of nut strikes or damaged nuts. Hull strikes could be used as a proxy to estimate the number of almonds with internal damage such as nut strikes or kernel necrosis which might later be unsellable (Table 1 and Table 2). Evidence of hemipteran probing and feeding has been used as a proxy for feeding damage in other systems. Nut strikes are expected to occur due to feeding injury from sucking insects where insects probe their mouthparts into fruits during feeding. In 2014, nut strikes on the four varieties due to *L. clypealis* was an average of 15%, while nut strikes from *L. zonatus* averaged 24.75%; in 2015, *L. zonatus* similarly had higher numbers of nut strikes than in controls.

In contrast to the relationship found between hull strikes and other kernel damage from *Leptoglossus* feeding, in other crops, the presence of holes, spots, or mouthpart stylet sheaths on a fruit has not always been found to translate directly into fruit damage. For example, Wiman et al. [35] reported that in blueberry, external fruit probing by *Halyomorpha halys* indicated by a high numbers of stylet sheaths had very little internal damage as measured necrosis and discoloration. However, in apples, the punctures by the mullein bug were related to internal damage [13], which is more similar to what was observed in this study.

Interestingly, some kernel necrosis was observed in control cages of all the varieties in both years. In 2014, the controls in each variety had few or no almonds with hull strikes yet had some level of kernel necrosis. A similar pattern was observed in 2015 as well. In cages with feeding by *Leptoglossus* there was a higher level of kernel necrosis than in controls. It should be noted that *Leptoglossus* feeding does not account for the kernel necrosis observed in the controls, but perhaps another agent such as a pathogen could be responsible. Studies such as the one herein with caged insects help to determine the damage symptoms which can be attributed to insect feeding. Similarly, a study of pistachios used caged bugs to determine which damage symptoms could be attributed to feeding [2]. Pathogens may be responsible for a percentage of kernel necrosis observed in controls in this study, but this would need to be investigated. The final damage parameter, shriveled kernels, was also observed in all the controls, similar to the pattern observed for kernel necrosis. It should again be noted that this is a natural type of damage which occurs in the crop, and it is not only induced by feeding of *L. clypealis* and *L. zonatus.* However, in *L. zonatus* cages, two varieties (Nonpareil and Monterey) had levels of shriveled kernels which exceeded the controls.

## 5. Conclusions

In summary, this study found that both *Leptoglossus* species, *L. clypealis* and *L. zonatus*, can have a significant impact on the production of the almond crop. Early season feeding resulted in substantial levels of almond drop in all four varieties, especially in the first four weeks of the study in April. The size of the almond and hull width did not directly relate to the level of almond drop observed; however, almond drop decreased as the shell hardened in May. All four varieties were vulnerable when infested with an equal number of adults. Kernel strikes were higher in the bug feeding cages than in the controls for both species. Kernel necrosis occurred in controls but was higher in cages with *Leptoglossus* spp.; the causative agent of almond kernel necrosis could be further investigated. In addition, shriveled kernels were at similar levels in *L. clypealis* cages as in controls but were higher than controls in two of the varieties with *L. zonatus.*

*L. clypealis* and *L. zonatus* are native insects feeding on an introduced crop (almonds) which is widely planted through the Central Valley of California. *L. clypealis* and *L. zonatus* have been reported in previous years as sporadic pests and *L. zonatus* appears to be increasing in abundance. Damage is significantly higher from the larger bug species, *L. zonatus*, than from *L. clypealis*. Currently, there are few control options for these insects, but investigations are underway of semiochemicals and other attractants which might be used in traps or lures to monitor or control these insects [7,36,37,38,39]. This study provides evidence that feeding by *L. clypealis* and *L. zonatus* can result in significant damage to almonds.

## Figures and Tables

**Table 1:** Final damage assessment in 2014 of almonds in four varieties following three treatments.

Variety	Damage Type	Control	*L. clypealis*	*L. zonatus*	χ^2^, *p* Value
Nonpareil	Hull strike	3% b	18% a	16% a	10.79, <0.001 *
	Kernel necrosis	3% b	12% a	14% a	7.15, <0.001 *
	Nut strike	2% b	25% a	31% a	24.25, <0.001 *
	Shriveled	6% a	3% a	6% a	1.20, 0.549
Fritz	Hull strike	0% b	17% a	18% a	11.54, <0.001 *
	Kernel necrosis	7% c	16% b	27% a	12.04, <0.001 *
	Nut strike	0% c	14% b	25% a	23.46, <0.001 *
	Shriveled	4% a	7% a	7% a	1.00, 0.607
Monterey	Hull strike	1% b	7% a	11% a	8.04, 0.012 *
	Kernel necrosis	13% b	13% b	31% a	11.37, <0.001 *
	Nut strike	1% b	11% a	20% a	16.93, <0.001 *
	Shriveled	12% b	7% b	27% a	14.13, <0.001 *
Carmel	Hull strike	0% b	6% a	12% a	12.0, <0.001 *
	Kernel necrosis	11% b	11% b	34% a	18.89, <0.001 *
	Nut strike	0% c	6% b	23% a	29.44, <0.001 *
	Shriveled	11% b	8% b	29% a	16.13, <0.001 *

Each cell represents the percent of almonds damaged in the final sample. Chi square χ^2^ goodness of fit was used to examine damage among the three treatments (in each row); an asterisk * indicates a significant difference. Different letters within a row are significantly different by Fisher’s exact test.

**Table 2:** Final damage assessment from 2015 of almonds in four varieties following three different treatments.

Variety	Damage Type	Control	*L. zonatus*	χ^2^, *p* Value
Nonpareil	Hull strike	9% b	43% a	22.23, <0.001 *
	Kernel necrosis	22% b	53% a	12.81, <0.001 *
	Nut strike	4% b	60% a	49.0, <0.001 *
	Shriveled	19% b	45% a	10.56, <0.001 *
Fritz	Hull strike	2% b	23% a	17.64, <0.001 *
	Kernel necrosis	12% b	48% a	21.60, <0.001 *
	Nut strike	0% b	20% a	25.00, <0.001 *
	Shriveled	4% a	9% a	2.88, 0.090
Monterey	Hull strike	0% b	11% a	11.0, <0.001 *
	Kernel necrosis	20% b	71% a	28.58, <0.001 *
	Nut strike	0% b	54% a	54.0, <0.001 *
	Shriveled	22% b	77% a	30.56, <0.001 *
Carmel	Hull strike	3% b	25% a	17.29, <0.001 *
	Kernel necrosis	15% b	40% a	11.36, <0.001 *
	Nut strike	11% b	51% a	25.81, <0.001 *
	Shriveled	20% a	25% a	0.56, 0.454

Each cell represents the percent of almonds damaged of the final sample. Chi square χ^2^ goodness of fit was used to examine damage among the treatments (in each row); an asterisk * indicates a significant difference. Different letters within a row represent a significant difference in treatments.

## Supplementary Materials

The following are available online at https://www.mdpi.com/2075-4450/10/10/333/s1, Table S1: The 2014 percent almond drop in four treatments in four almond varieties. CO = control, PU = punctured, CL = *L. clypealis*, and ZO = *L. zonatus*. n/a = no observation in that week, Table S2: Comparison of weekly almond drop from feeding by *L. clypealis* within each variety in 2014. Percent almond drop is compared in each two weeks of observations by Fisher’s exact tests. *p* < 0.05 is significant and shown in bold. (-) indicates no comparisons due to no observation in week 1 or 7 in Monterey or Carmel varieties. Table S3: Comparison of weekly almond drop from feeding by *L. zonatus* within each variety in 2014. Each comparison is between percent almond drop in two weeks of observations. Comparisons are by Fisher’s exact tests and *p* < 0.05 is considered significant and shown in bold. (-) indicates no comparisons due to no observation in week 1 or 7 in Monterey or Carmel. Table S4: The 2015 weekly percent almond drop in four treatments in four almond varieties. The date corresponds to the observation week in the study.CO = control, PU = punctured, CL = L. clypealis, and ZO = L. zonatus. n/a = no observation in that week. Table S5: Weekly almond drop by *L. zonatus* within each variety in 2015. Each comparison is percent almond drop in between two weeks (Fisher’s exact tests; *p* < 0.05). (-) indicates no observation week 3 in Nonpareil and Fritz, and week 1 in Monterey or Carmel.
