# Supplementary material for: Assessing Feeding Damage from Two Leaffooted Bugs, Leptoglossus clypealis Heidemann and Leptoglossus zonatus (Dallas) (Hemiptera: Coreidae), on Four Almond Varieties
Source: Insects. 2019 Oct 7;10(10):333. doi: 10.3390/insects10100333 (PMC6835505; doi:10.3390/insects10100333)
Supplement: Supplementary file 1 [file insects-10-00333-s001.pdf]

Table S1. The 2014 weekly percent almond drop in four treatments in four almond varieties. The date corresponds to the observation week in the study. CO=control, PU=punctured, CL=*L. clypealis*, and ZO=*L. zonatus*. n/a = no observation in that week.

		**Nonpareil**				**Fritz**				**Monterey**				Carmel			
**week**	**Date**	**CO**	**PU**	**CL**	**ZO**	**CO**	**PU**	**CL**	**ZO**	**CO**	**PU**	**CL**	**ZO**	**CO**	**PU**	**CL**	**ZO**
W1	Mar 24	5	86.25	15	20	2.5	91.25	15	25	n/a	n/a	n/a	n/a	n/a	n/a	n/a	n/a
W2	Apr 2	14.46	82.46	60	66.67	33.75	93.48	5	75	20.41	76.52	52.94	93.75	17.22	93.75	28.57	40
W3	Apr 9	2.5	85.06	50	100	12.5	84.71	63.16	95	28.94	91.11	38.89	95	41.93	78.1	0	75
W4	Apr 14	6.25	91.25	15	45	0	82.5	25	10	0	2.64	15	0	0	10.83	15	5
W5	Apr 28	0	29.93	10	10	1.25	29.41	5	11.11	0	87.5	5	90	1.25	76.25	100	80
W6	May 15	0	0	0	0	0	27.54	0	33.33	0	7.59	0	60	0	6.25	100	20
W7	May 21	0	5	10	0	8.88	6.66	5.26	5	n/a	n/a	n/a	n/a	n/a	n/a	n/a	n/a
W8	May 28	0	0	0	0	0	0.025	0	0	0	0	0	0	3.3	5	0	5.26

Table S2. Comparison of weekly almond drop from feeding by *L. clypealis* within each variety in 2014. Percent almond drop is compared in each two weeks of observations by Fisher's exact tests. P<0.05 is significant and shown in bold. (-) indicates no comparisons due to no observation in week 1 or 7 in Monterey or Carmel varieties.

**Weeks compared**	Nonpareil	Fritz	Monterey	Carmel
W1-W2	**0.008**	0.605	**--**	**----**
W1-W3	**0.023**	**0.003**	**--**	**--**
W1-W4	1.000	0.695	**--**	**--**
W1-W5	1.000	0.605	**--**	**--**
W1-W6	0.231	0.231	**--**	**--**
W1-W7	1.000	0.605	--	--
W1-W8	0.231	0.231	--	--
W2-W3	0.546	**<0.001**	0.366	**0.020**
W2-W4	**0.00**	0.182	**0.010**	**0.451**
W2-W5	**0.002**	1.000	**<0.001**	**<0.001**
W2-W6	**<0.001**	1.000	<0.001	**<0.001**
W2-W7	**0.002**	1.000	--	--
W2-W8	**<0.001**	1.000	<0.001	**0.020**
W3-W4	**0.041**	**0.025**	0.155	0.231
W3-W5	**0.014**	**<0.001**	**0.020**	**<0.001**
W3-W6	**<0.001**	**<0.001**	**0.003**	**<0.001**
W3-W7	**0.012**	**<0.001**	--	--
W3-W8	**<0.001**	**<0.001**	**0.003**	1.000
W4-W5	1.000	0.101	0.605	**<0.001**
W4-W6	0.231	**0.024**	0.231	**<0.001**
W4-W7	1.000	0.101	--	--
W4-W8	0.231	**0.024**	0.231	0.231
W5-W6	0.487	1.000	1.000	1.000
W5-W7	1.000	1.000	--	--
W5-W8	0.487	1.000	1.000	**<0.001**
W6-W7	0.487	1.000	--	--
W6-W8	1.000	1.000	1.000	**<0.001**
W7-W8	0.487	1.000	--	--

Table S3. Comparison of weekly almond drop from feeding by *L. zonatus* within each variety in 2014. Each comparison is between percent almond drop in two weeks of observations. Comparisons are by Fisher's exact tests and $p<0.05$ is considered significant and shown in bold. (-) indicates no comparisons due to no observation in week 1 or 7 in Monterey or Carmel.

**Weeks compared**	**Variety**			
	**Nonpareil**	**Fritz**	**Monterey**	**Carmel**
W1-W2	**0.005**	**0.002**	--	--
W1-W3	**0.005**	**0.000**	--	--
W1-W4	0.108	0.408	--	--
W1-W5	0.422	0.408	--	--
W1-W6	0.053	0.731	--	--
W1-W7	0.053	0.101	--	--
W1-W8	0.053	**0.024**	--	--
W2-W3	**0.008**	0.182	1.000	**0.032**
W2-W4	0.341	**<0.001**	**<0.001**	**0.020**
W2-W5	**0.008**	**<0.001**	1.000	**0.003**
W2-W6	**<0.001**	**0.025**	**<0.020**	**0.030**
W2-W7	**<0.001**	**<0.001**	--	--
W2-W8	**<0.001**	**<0.001**	**<0.001**	**0.020**
W3-W4	**<0.001**	**<0.001**	**<0.001**	**0.100**
W3-W5	**<0.001**	**<0.001**	1.000	**<0.001**
W3-W6	**<0.001**	**<0.001**	**0.020**	**<0.001**
W3-W7	**<0.001**	**<0.001**	--	--
W3-W8	**<0.001**	**<0.001**	**<0.001**	**<0.001**
W4-W5	**0.017**	1.000	**<0.001**	<0.001
W4-W6	**<0.001**	0.127	**<0.001**	0.342
W4-W7	**<0.001**	1.000	--	--
W4-W8	**<0.001**	0.487	1.000	1.000
W5-W6	0.487	0.127	<0.065	<0.001
W5-W7	0.487	1.000	--	--
W5-W8	0.487	0.127	**<0.001**	<0.001
W6-W7	1.000	**0.044**	--	--
W6-W8	1.000	**0.008**	**<0.001**	0.194
W7-W8	1.000	1.000	--	--

Table S4. The 2015 weekly percent almond drop in four treatments in four almond varieties. The date corresponds to the observation week in the study. CO=control, PU=punctured, CL=*L. clypealis*, and ZO=*L. zonatus*. n/a = no observation in that week.

		Nonpareil			Fritz			Monterey			Carmel		
**week**	**Date**	**CO**	**PU**	**ZO**	**CO**	**PU**	**ZO**	**CO**	**PU**	**ZO**	**CO**	**PU**	**ZO**
W1	Mar 24	2.56	92.86	91.30	14.74	62.65	61.90	n/a	n/a	n/a	n/a	n/a	n/a
W2	Apr 2	0.00	73.02	100	26.76	93.85	94.12	8.77	100.00	95	0	67.14	0
W3	Apr 9	n/a	n/a	n/a	n/a	n/a	n/a	0.00	86.21	100	0	32.14	9
W4	Apr 13	15.00	48.78	60.00	12.50	50.00	42.11	35.29	66.67	75	0	18.52	20
W5	Apr 21	0.00	20.69	100	16.13	46.88	70.00	5.41	34.48	80	0	36.36	90
W6	Apr 28	9.09	10.34	80.00	0.00	37.84	65.00	5.13	38.24	65	3.57	3.70	0
W7	May 5	7.50	0.00	13.33	3.03	5.88	0.00	0.00	0.00	5.26	0	0.00	28.57
W8	May 12	0.00	0.00	6.67	0.00	2.94	0.00	5.41	0.00	0	0	3.33	0
W9	May 21	0.00	0.00	0.00	5.56	0.00	28.57	0.00	0.00	0	0	0.00	0

Table S5. Weekly almond drop by *L. zonatus* within each variety in 2015. Each comparison is percent almond drop in between two weeks (Fisher's exact tests; p<0.05). (-) indicates no observation week 3 in Nonpareil and Fritz, and week 1 in Monterey or Carmel.

**Weeks compared**	**Nonpareil**	**Fritz**	**Monterey**	**Carmel**
W1-W2	0.244	**0.010**	--	--
W1-W3	--	--	--	--
W1-W4	0.065	0.228	--	--
W1-W5	0.244	0.531	--	--
W1-W6	0.661	0.757	--	--
W1-W7	**<0.001**	**<0.001**	--	--
W1-W8	**<0.001**	**<0.001**	--	--
W1-W9	**<0.001**	0.068	--	--
W2-W3	--	--	1.000	0.487
W2-W4	**0.003**	**<0.001**	0.101	0.106
W2-W5	1.000	**0.049**	0.342	**<0.001**
W2-W6	0.106	**0.044**	**<0.001**	1.000
W2-W7	**<0.001**	**<0.0001**	**<0.001**	**0.020**
W2-W8	**<0.001**	**<0.001**	**<0.001**	1.000
W2-W9	**<0.001**	**<0.001**	**<0.001**	1.000
W3-W4	--	--	**0.024**	0.661
W3-W5	--	--	0.106	**<0.001**
W3-W6	--	--	**0.008**	0.487
W3-W7	--	--	**<0.001**	0.235
W3-W8	--	--	**<0.001**	0.487
W3-W9	--	--	**<0.001**	0.487
W4-W5	**0.003**	0.679	1.000	**<0.001**
W4-W6	0.301	0.205	0.731	0.106
W4-W7	**0.008**	**0.003**	**<0.001**	0.716
W4-W8	**<0.001**	**0.003**	**<0.001**	0.106
W4-W9	<0.001	0.531	**<0.001**	0.106
W5-W6	0.106	1.000	0.480	**<0.001**
W5-W7	**<0.001**	**<0.001**	**<0.001**	**0.0002**
W5-W8	**<0.001**	**<0.001**	**<0.001**	**0.0001**
W5-W9	**<0.001**	**0.026**	**<0.001**	**0.0001**
W6-W7	**<0.001**	**<0.001**	**<0.001**	**0.0202**
W6-W8	**<0.001**	**<0.001**	**<0.001**	1.0000
W6-W9	**<0.001**	0.056	**<0.001**	1.0000
W7-W8	0.605	1.000	1.000	**0.0202**
W7-W9	0.238	**0.020**	1.000	**0.0202**
W8-W9	1.0000	**0.020**	1.000	1.0000
